# *gyrA* and *parC* mutations in fluoroquinolone-resistant *Neisseria gonorrhoeae* isolates from Kenya

**DOI:** 10.1186/s12866-019-1439-1

**Published:** 2019-04-08

**Authors:** Mary Wandia Kivata, Margaret Mbuchi, Fredrick Lunyagi Eyase, Wallace Dimbuson Bulimo, Cecilia Katunge Kyanya, Valerie Oundo, Simon Wachira Muriithi, Ben Andagalu, Wilton Mwema Mbinda, Olusegun O. Soge, R. Scott McClelland, Willy Sang, James D. Mancuso

**Affiliations:** 1 US Army Medical Research Directorate-Africa, P.O Box 606, 00621, Village Market, Nairobi, Kenya; 2 Institute for Biotechnology Research, Jomo Kenyatta University of Agriculture and Technology (JKUAT), P.O Box 62,000-00200, Thika, Kenya; 3 Department of Biological and Physical Science, Karatina University (KarU), P.O Box 1957-10101, Karatina, Kenya; 4 Kenya Medical Research Institute (KEMRI), P. O. Box 54840-00200, Nairobi, Kenya; 5 Department of Global Health and Medicine, University of Washington, 325 9th Avenue, Box 359931, Seattle, WA 98104 USA; 6 Department of Biochemistry, School of Medicine, University of Nairobi, P.O. Box 30197, GPO, 00100, Nairobi, Kenya

**Keywords:** Fluoroquinolones, Antimicrobial resistance (AMR), *Neisseria gonorrhoeae*, Mutation, Quinolone resistant determining regions (QRDR)

## Abstract

**Background:**

Phenotypic fluoroquinolone resistance was first reported in Western Kenya in 2009 and later in Coastal Kenya and Nairobi. Until recently gonococcal fluoroquinolone resistance mechanisms in Kenya had not been elucidated. The aim of this paper is to analyze mutations in both *gyrA* and *parC* responsible for elevated fluoroquinolone Minimum Inhibitory Concentrations (MICs) in *Neisseria gonorrhoeae* (GC) isolated from heterosexual individuals from different locations in Kenya between 2013 and 2017.

**Methods:**

Antimicrobial Susceptibility Tests were done on 84 GC in an ongoing Sexually Transmitted Infections (STI) surveillance program. Of the 84 isolates, 22 resistant to two or more classes of antimicrobials were chosen for analysis. Antimicrobial susceptibility tests were done using E-test (BioMerieux) and the results were interpreted with reference to European Committee on Antimicrobial Susceptibility Testing (EUCAST) standards. The isolates were sub-cultured, and whole genomes were sequenced using Illumina platform. Reads were assembled de novo using Velvet, and mutations in the GC Quinolone Resistant Determining Regions identified using Bioedit sequence alignment editor. Single Nucleotide Polymorphism based phylogeny was inferred using RaxML.

**Results:**

Double GyrA amino acid substitutions; S91F and D95G/D95A were identified in 20 isolates. Of these 20 isolates, 14 had an additional E91G ParC substitution and significantly higher ciprofloxacin MICs (*p* = 0.0044*). On the contrary, norfloxacin MICs of isolates expressing both GyrA and ParC QRDR amino acid changes were not significantly high (*p* = 0.82) compared to MICs of isolates expressing GyrA substitutions alone. No single GyrA substitution was found in the analyzed isolates, and no isolate contained a ParC substitution without the simultaneous presence of double GyrA substitutions. Maximum likelihood tree clustered the 22 isolates into 6 distinct clades.

**Conclusion:**

Simultaneous presence of amino acid substitutions in ParC and GyrA has been reported to increase gonococcal fluoroquinolone resistance from different regions in the world. Our findings indicate that GyrA S91F, D95G/D95A and ParC E91G amino acid substitutions mediate high fluoroquinolone resistance in the analyzed Kenyan GC.

## Background

The absence of a gonococcal vaccine reduces preventive measures for gonorrhea and antibiotics remain the only option for management of gonococcal infections. While initially sensitive to new antibiotics, some strains of *Neisseria gonorrhoeae* (GC) develop single or multiple resistance mechanisms to antibiotics. Gonococcal mechanisms of resistance can be plasmid mediated or result from different types of mutations in chromosomal DNA, as well as recombination processes [1–4]. Since the 1970s, a small number of studies have reported both chromosomal and plasmid mediated gonococcal penicillin and tetracycline resistance in Kenya [5–9]. These reports led to the introduction of fluoroquinolones as the first line of drugs for gonorrhea treatment in 1993 [10]. Subsequently, fluoroquinolone resistance was reported in western Kenya in 2009 [11], Coastal Kenya in 2011 and 2012 [12, 13], and Nairobi in 2012 [12]. These findings formed the basis for revision of national treatment guidelines in 2013. In the revised guidelines, ceftriaxone and cefixime replaced fluoroquinolones as first line drugs for gonorrhea treatment. Recently, investigators have begun to explore the molecular mechanisms causing fluoroquinolone resistance in Kenya. A 2018 publication focused on sex workers and Men who have Sex with Men (MSM) from Coastal Kenya found that S91F, D95G/A, and F504 L GyrA amino acid substitutions were associated with fluoroquinolone resistance [14].

Fluoroquinolones block DNA replication by inhibiting the enzymes DNA gyrase (topoisomerase II) and topoisomerase IV [15]. DNA gyrase catalyzes the untwisting of DNA molecules during DNA replication, and consists of two type A subunits and two type B subunits encoded by *gyrA* and *gyrB* genes [16]. Topoisomerase IV consists of two type C subunits and two type E subunits encoded by *parC* and *parE* genes. This enzyme is involved in the decatenation of covalently closed circular DNA molecules during DNA replication [15]. DNA-enzyme-fluoroquinolone-complex inhibits movement of the replication fork; a structure formed by organization of replication proteins and disrupts bacterial DNA replication. Gradual accumulation of point mutations in *gyrA* and *parC* Quinolone Resistant Determining Region (QRDR) leads to amino acid substitutions, which alter the three-dimensional structure of the target protein [17]. Alteration of the target protein structure reduces fluoroquinolone-target enzyme binding affinity, leading to resistance in gonococci [4, 18]. Gonoccocal QRDR region lies between amino acids 55–110 and 56–140 in GyrA and ParC respectively [18]. QRDR amino acid substitutions linked to gonococcal fluoroquinolone resistance include substitutions at S91 by F or Y, and D95 in GyrA protein [17, 19]. The GyrA mutations initiate fluoroquinolone resistance in GC, while additional accumulation of substitutions in ParC at G85, D86, S87, S88, Q9l and R116 increases the resistance [4]. In addition to mutations in the QRDR, an in-vitro study by Tanaka et al., [20] suggested that mutations in PorB can cause reduced drug accumulation, which can contribute further to the development of gonoccocal fluoroquinolone resistance.

The aim of this study was to analyze amino acid changes in GyrA and ParC QRDRs responsible for gonococcal fluoroquinolone resistance and determine the genetic diversity of multidrug resistant Kenyan GC. In a previous correlation study carried out in Japan, a significant increase in fluoroquinolone MICs (*P* < 0.01 for norfloxacin, *P* = 0.058 for ofloxacin, and *P* < 0.05 for ciprofloxacin) was observed in GC strains which expressed amino acid changes in both GyrA and ParC compared to those of strains expressing GyrA substitutions only [21]. This study therefore sought to further examine the correlation between GyrA and ParC amino acid changes and the level of fluoroquinolone resistance among GC isolated from heterosexual individuals from different geographical regions of Kenya between 2013 and 2017.

## Results

### Clinical and laboratory study population characteristics

The current study is a retrospective laboratory-based study nested in an ongoing Sexually Transmitted Infections (STI) surveillance program under Armed Forces Health Surveillance at the US Army Medical Research Directorate-Africa. A total of 583 symptomatic cases comprising of 332 males and 251 females were enrolled from four geographic locations in Kenya (Nairobi, Coastal Kenya, Nyanza, and Rift Valley) between 2013 and 2017. All presented with urethral discharge (males) or cervical discharge (females) accompanied by symptoms described in Table 1.

**Table 1:** Clinical and laboratory study population characteristics

	Male	Female	Total
Gender	332 (56.9%)	251 (43.1)	583
Gram stain^a^	168 (67.7%)	80 (32.3)	248
Aptima Combo −2®^b^	205 (77.9%)	58 (22.1)	263
GC isolates	73 (86.9%)	11 (13.1%)	84
Symptoms	Positive	Negative	
Dysuria	463 (79.4%)	120 (20.6%)	583
Backache	274 (47%)	309 (53%)	583
Lower abdominal pain	388 (66.6%)	195 (33.4%)	583
Fever	261 (44.8%)	322 (55.2%)	583
Itchy genitalia	342 (58.7%)	241 (41.3%)	583

^a^Positive for gram negtive diplococci by gram stain

^b^Positive for *Neisseria gonorrhoeae* (GC) by Aptima Combo −2®

Eighty four *Neisseria gonorrhoeae* (GC) isolates were successfully obtained from 73 males and 11 females and their susceptibility to a panel of ten drugs (Ceftriaxone, Cefixime, Azithromycin, Ciprofloxacin, Norfloxacin, Spectinomycin, Tetracycline, Doxycycline, Penicillin and Gentamicin) carried out using the E-test (bioMerieux) method. There was no difference in the prevalence of multidrug resistance among isolates from the different regions. Of the 84, 22 multi-drug resistant GC isolates (2 females and 20 males) were chosen for analysis.

### Antibiotic susceptibilities of studied isolates

Of the 22 isolates selected for sequencing 20 were ciprofloxacin resistant (MICs > 0.06 mg/L), whereas 2 were susceptible. All isolates were penicillin resistant (MICs> 0.06 mg/L) while 16 were tetracycline resistant (MICs> 1 mg/L). Low level azithromycin resistance (MICs> 0.05-2 mg/L) was observed in 7 isolates. None of the isolates were resistant to cefixime, ceftriaxone, or spectinomycin.

### Single nucleotide polymorphisms (SNPs) based phylogeny

**Fig. 1:**
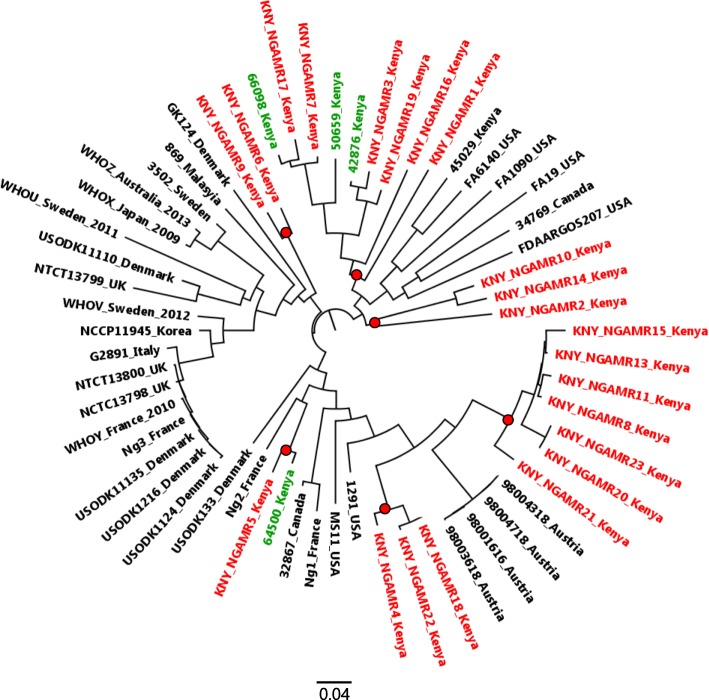
Maximum likelihood SNP phylogenetic tree inferred using RaxML. The 22 study isolates (KNY) in red and 4 Kenyan isolates (in green) downloaded from NCBI (64500, 66098, 50659, and 42876) and previously obtained from MSM population clustered closely together into six distinct clades shown in red circles

Sixty sequences; 22 study genomes and 38 additional comparison genomes obtained from varied geographical regions, were mapped to *N gonorrhoeae* NCCP11945 (GenBank accession number CP001050) genome and SNPs identified through CSI phylogeny pipeline. A total of 24,143 SNPs were detected and were used to create a Maximum Likelihood tree using RaxML (Fig. 1). Among the analyzed Kenya GC, Isolates KNY_NGAMR1 and KNY_NGAMR2 were the most genetically diverse (5569SNPs), while KNY_NGAMR20 and KNY_NGAMR23 were closely related with a SNP difference of 10. Based on SNP phylogenetic tree, the 22 study isolates, and 4 isolates obtained from MSMs from Coastal Kenya in a recent study [14], clustered closely together into six distinct clades (Fig. 1). The close clustering shows that Kenyan isolates have evolved into their own lineages. However, there was no region based clustering observed among the 22 isolates.

### Fluoroquinolone resistance determinants

**Fig. 2:**
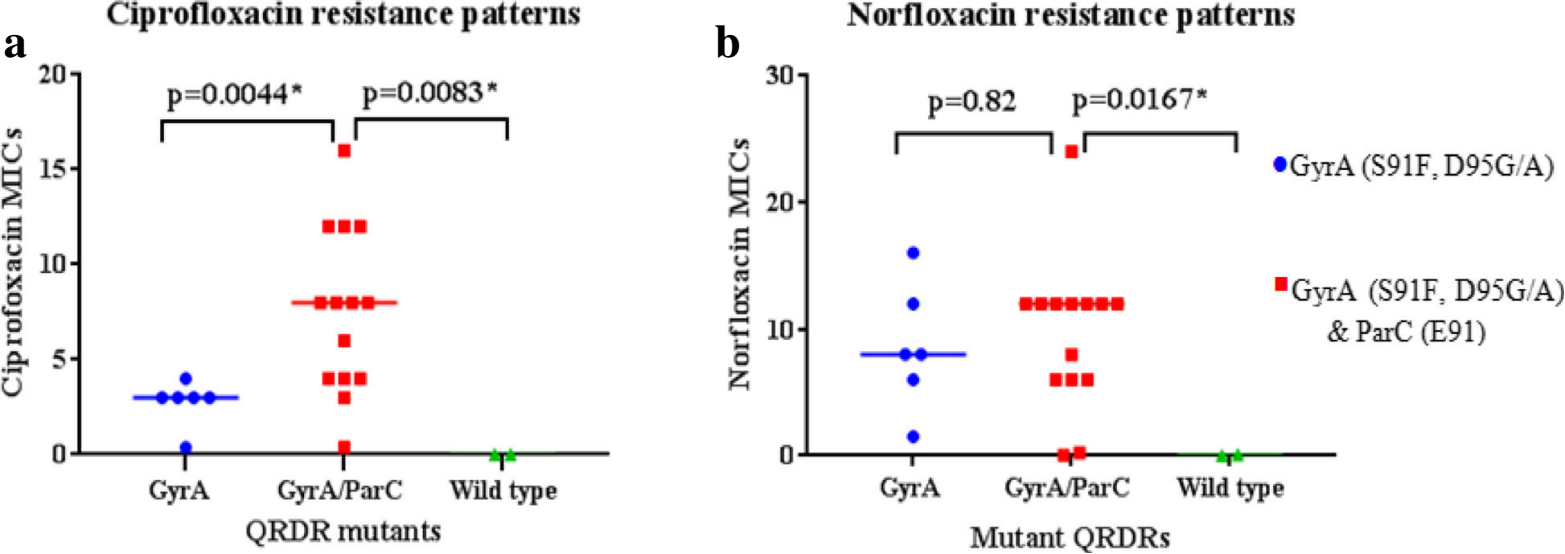
Ciprofloxacin (**a**) and Norfloxacin (**b**) MICs of GC isolates expressing QRDR amino acid substitutions. The central bars across each group of points locates the median for that group

Of the 22 isolates, 2 had wild-type QRDRs, 6 had only *gyrA* QRDR mutations, and the remaining 14 had both *gyrA* and *parC* QRDR mutations. Double GyrA amino acid substitutions; S91F and D95G/A (corresponding to C272T, A284G or A284C *gyrA* mutations respectively) were identified in 20 of the 22 isolates. The 20 isolates were all ciprofloxacin-resistant (MICs > 0.06 mg/L) (Table 2). Of these 20 isolates, 14 had ParC E91G (corresponding to A272G *parC* mutation) amino acid substitution. The remaining 2 isolates (KNY_NGAMR2 and KNY_NGAMR4) which were susceptible to ciprofloxacin (MICs ≤0.03 mg/L) lacked both *gyrA* and *parC* QRDR mutations (Fig. 2a, and Table 2).

**Table 2:** Ciprofloxacin (CIP) and norfloxacin (NOR) susceptibility data for the 22 GC strains with details of GyrA, ParC, MtrR and PorB amino acid substitutions, and *mtrR* promoter mutation

Isolate ID	MIC(mg/L)		Mutations									
			*Gyr A*		*Par C*	*MtrR*			A13deletion	*PorB*		
	CIP	NOR	S91	D95	E91	A39	G45	H105		A121	G120	N122
KNY_NGAMR1	0.38	1.5	F	G								
KNY_NGAMR2	0.016^a^	0.094					D		A deleted		D	
KNY_NGAMR3	3	6	F	G	G	T						
KNY_NGAMR4	0.006^a^	0.023				T				S		K
KNY_NGAMR5	4	6	F	G	G			Y				
KNY_NGAMR6	12	12	F	G	G	T						
KNY_NGAMR7	8	12	F	G	G					S		K
KNY_NGAMR8	3	6	F	A		T						
KNY_NGAMR9	12	0.24	F	A	G	T						
KNY_NGAMR10	8	12	F	G	G	T						
KNY_NGAMR11	3	8	F	A		T						
KNY_NGAMR13	3	12	F	A		T						
KNY_NGAMR14	16	24	F	G	G	T				S		K
KNY_NGAMR15	4	16	F	A		T						
KNY_NGAMR16	8	6	F	G	G	T						
KNY_NGAMR17	6	12	F	G	G			Y				
KNY_NGAMR18	0.38	0.047	F	G	G	T						
KNY_NGAMR19	4	8	F	G	G	T						
KNY_NGAMR20	8	12	F	A	G	T						
KNY_NGAMR21	3	8	F	A		T						
KNY_NGAMR22	4	12	F	G	G	T						
KNY_NGAMR23	12	12	F	A	G	T						

GyrA S91F and D95G/A amino acid substitutions were found in 20 ciprofloxacin resistant isolates. Of the 20 isolates, 14 had an additional ParC E91G substitution. Two ciprofloxacin susceptible isolates lacked the QRDR mutations associated with fluoroquinolone resistance

^a^**-**ciprofloxacin susceptible

Ciprofloxacin MICs for GC isolates with double GyrA QRDR amino acid substitutions, and for those with both GyrA and ParC QRDR amino acid substitutions were significantly higher compared to MICs of isolates expressing wild-type GyrA and ParC QRDRs (*p* = 0.0357* and *p* = 0.0083* respectively). Additionally, compared to the ciprofloxacin MICs of GC isolates with double GyrA QRDR amino acid substitutions alone, the MICs of GC isolates with an additional ParC amino acid substitution were significantly higher (*p* = 0.0044*) (Fig. 2a). Norfloxacin MICs of GC isolates expressing both GyrA and ParC QRDR amino acid substitutions were significantly different from those of GC isolates with wild-type GyrA and ParC QRDRs (*p* = 0.0167*). There was no significant difference between norfloxacin MICs of GC isolates expressing double GyrA QRDR amino acid substitutions and those expressing both GyrA and ParC QRDR amino acid substitutions (*p* = 0.82) (Fig. 2b).

No single GyrA amino acid substitutions were found in the QRDR of the analyzed isolates and there was no isolate containing a ParC QRDR amino acid substitution without the simultaneous presence of double GyrA QRDR amino acid substitutions. No amino acid changes were found at G85, D86, S87, S88, and R116 of ParC as reported by other studies from other countries [4, 22]. Additional amino acid substitutions found outside the QRDR regions in both GyrA and ParC and which have not yet been associated with gonococcal fluoroquinolone resistance are; GyrA (M250I), and ParC (A156T, P289L, V359 M, I384V, A435V, F479 L, and I596V).

The *mtrR* gene encodes a repressor protein which represses expression of the *mtrCDE* operon encoding an efflux pump. Mutations in *mtrR* gene and its promoter have been associated with fluoroquonolone resistance in gonococci [23, 24]. Polymorphism analysis of the *mtrR* gene and its promoter showed that; only one isolate (KNY_NGAMR2) had the Adenine deletion in the 13 bp inverted repeat region between the − 10 and − 35 hexamers of the *mtrR* gene promoter while 20 isolates had A39T (17 isolates), G45D (1 isolate) and H105Y (2 isolates) amino acid substitutions in MtrR (Table 2). There were no cases of double amino acid substitutions as each MtrR mutant isolate had only one of the three amino acid substitutions. One isolate (KNY_NGAMR2) which lacked QRDR mutations had very low MICs for both drugs and had both G45D MtrR substitution and Adenine 13 deletion.

PorinB (PorB) is a protein which mediates influx of compounds into bacterial cells. Analysis of PorB protein at positions 120, 121 and 122 showed that 4 isolates (KNY_NGAMR2, KNY_NGAMR4, KNY_NGAMR7, and KNY_NGAMR17) had amino acid substitutions in PorB that have been associated with reduced drug permeability in bacterial cells. There was no observable relation between substitutions in the PorB and fluoroquonolone MICs (Table 2).

## Discussion

High prevalence of ciprofloxacin resistance by EUCAST breakpoints [25] (90.3%, MIC > 0.06 mg/L) was observed in the 84 GC isolates obtained in the STI Surveillance study. Simultaneous presence of amino acid substitutions in both GyrA and ParC QRDRs was associated with significantly higher ciprofloxacin and norfloxacin MICs when compared to MICs of isolates with wild-type QRDRs. Several studies have shown that GyrA amino acid substitutions initiate fluoroquinolone resistance in GC, while additional accumulation of substitutions in the ParC at G85, D86, S87, S88, Q9l and R116 elevate the resistance [4]. Our findings are in agreement with this previous observation as the ciprofloxacin MICs of GC isolates which had amino acid substitutions in both GyrA and ParC QRDRs were significantly higher compared to both the MICs of GC isolates with only double GyrA mutations and those with wild-type QRDRs. Double GyrA amino acid substitutions (S91F and D95G/A) were recently identified and associated with fluoroquinolone resistant GC isolated from MSM in Coastal Kenya [14].

There was no significant increase in norfloxacin MICs in GC isolates expressing both GyrA and ParC QRDR amino acid substitutions, when compared to MICs of isolates expressing double GyrA mutations alone. Ciprofloxacin and norfloxacin differ at position N1 of the fluoquinolone acid core, where ciprofloxacin has a cyclopropane ring and norfloxacin has an ethyl group [26, 27]. This structural difference could have contributed to the different MIC patterns observed between the two drugs in this study. Although our study was limited to the 22 sequenced GC isolates the significant difference in norfloxacin MICs observed between GC isolates expressing both GyrA and ParC QRDR amino acid substitutions and those expressing wild-type QRDRs indicate that the triple QRDR amino acid substitutions could be involved in mediating norfloxacin resistance in the Kenyan GC.

An in-vitro study by Tanaka et al., [20] suggested that reduced drug accumulation in cells contribute to the development of fluoroquinolone resistance in gonococci. Reduced drug accumulation can result from an active efflux system or reduced drug influx [28]. Mutations in the promoter or encoding region of the *mtrR* gene, which leads to over expression of the *mtrCDE* operon has been associated with resistance to antibacterial agents [29]. Common mutations in *mtrR* implicated in increased drug efflux in GC include the: adenine deletion (A-) in the 13 bp inverted repeat region between the − 10 and − 35 hexamers of the *mtrR* gene promoter, and A39T, G45D, and H105Y amino acid substitutions in MtrR [30]. Warner and Shafer, [30] highlighted that the location of A39T substitution in MtrR can have significant effect in binding of the repressor protein to its target thereby causing de-repression of *mtrCDE* operon and consequently increased drug efflux. No relationship was observed between MtrR A39T substitution and fluoroquinolone resistance in the present study.

Reduced drug permeation through the porinB protein in GC has been associated with substitutions with charged amino acids at G120 and A121, and deletions of A121 and N122 in the PorB [31]. Although 4 isolates had amino acid substitutions in PorB that have been associated with reduced drug permeability in bacterial cells, this study did not find evidence for the contribution of reduced cell permeability and drug efflux towards fluoroquinolone resistance in analyzed GC isolates.

Mutations in L4 and L22 ribosomal proteins, and in 23 s rRNA genes known to cause high level azithromycin resistance were not identified in any of the study isolates. The low level of azithromycin resistance observed in 7 of the 22 isolates could be as a result of an active efflux MtrCDE pump or from reduced drug influx caused by a mutated PorB.

The 22 isolates obtained from heterosexual population formed 6 phylogenetically distinct clusters, three of which contained GC isolates recovered from MSM population from Coastal Kenya [14].

## Conclusion

Our study reports E91G amino acid substitution in ParC of fluoroquinolone resistant GC isolates from Kenya. The findings indicate that simultaneous presence of GyrA S91F, D95G/D95A, and ParC E91G amino acid substitutions mediate the high fluoroquinolone resistance observed in the analyzed Kenyan GC. Clustering observed in the Kenyan isolates indicate that they are closely related genetically and are evolving into their own distinct lineages.

## Methods

### Study design and study isolates

The current study is a retrospective laboratory-based study nested in an ongoing STI surveillance program under Armed Forces Health Surveillance at the US Army Medical Research Directorate -Africa. Study isolates were obtained as part of the study entitled “A surveillance study of antimicrobial susceptibility profiles of *Neisseria gonorrhoeae* isolates from patients seeking treatment in selected clinics in Kenya” Walter Reed Army Institute of Research (WRAIR) Human Subject Protection Board (HSPB) Protocol l#1743/Kenya Medical Research Institute Scientific and Ethics Review Unit (SERU) #1908.

Symptomatic males and females were enrolled from four geographic locations in Kenya (Nairobi, Coastal Kenya, Nyanza, and Rift Valley) between 2013 and 2017. Eighty four *Neisseria gonorrhoeae* (GC) isolates were successfully obtained and their susceptibility to a panel of ten drugs (Ceftriaxone, Cefixime, Azithromycin, Ciprofloxacin, Norfloxacin, Spectinomycin, Tetracycline, Penicillin and Gentamicin) carried out using the E-test (BioMerieux) method. GC isolates found to be resistant to more than two classes of drugs were classified as multidrug resistant. Twenty two archived and viable GC isolates resistant to two or more classes of antimicrobial agents were chosen for analysis (Table 3).MIC breakpoints for ciprofloxacin resistance were set at susceptible ≤0.003 mg/L, and resistant > 0.06 mg/L, based on the European Committee on Antimicrobial Susceptibility Testing (EUCAST) version 8.0, 2018.

**Table 3:** Isolate multi-drug resistance patterns

Isolate ID	Year of isolation	Resistance patterns						
		CFM	CRO	PEN	SPT	AZM	CIP	TET
KNY_NGAMR1	2015	S	S	R	S	S	R	S
KNY_NGAMR2	2015	S	S	R	S	R	S	S
KNY_NGAMR3	2015	S	S	R	S	S	R	R
KNY_NGAMR4	2016	S	S	R	S	S	S	R
KNY_NGAMR5	2016	S	S	R	S	S	R	R
KNY_NGAMR6	2017	S	S	R	S	S	R	S
KNY_NGAMR7	2014	S	S	R	S	R	R	R
KNY_NGAMR8	2013	S	S	R	S	S	R	S
KNY_NGAMR9	2016	S	S	R	S	S	R	S
KNY_NGAMR10	2016	S	S	R	S	S	R	R
KNY_NGAMR11	2016	S	S	R	S	S	R	R
KNY_NGAMR13	2014	S	S	R	S	S	R	R
KNY_NGAMR14	2015	S	S	R	S	S	R	R
KNY_NGAMR15	2014	S	S	R	S	R	R	R
KNY_NGAMR16	2015	S	S	R	S	S	R	R
KNY_NGAMR17	2015	S	S	R	S	R	R	R
KNY_NGAMR18	2015	S	S	R	S	R	R	R
KNY_NGAMR19	2015	S	S	R	S	R	R	S
KNY_NGAMR20	2015	S	S	R	S	S	R	R
KNY_NGAMR21	2016	S	S	R	S	S	R	R
KNY_NGAMR22	2016	S	S	R	S	R	R	R
KNY_NGAMR23	2017	S	S	R	S	S	R	R

MIC breakpoints were based on the European Committee on Antimicrobial Susceptibility Testing (EUCAST) version 8.0, 2018.CRO: Ceftriaxone CFM: cefixime, PEN: penicillin, SPT: spectinomycin, AZM: azithromycin, CIP: ciprofloxacin, and TET: tetracycline. S: susceptible MIC and R: resistance MIC. There are no EUCAST MIC breakpoints for norfloxacin, gentamycin and doxycycline

### Ethical consideration

Permission to carry out the study was obtained from both Kenya Medical Research Institute Scientific and Ethics Review Unit (SERU) and Walter Reed Army Institute of Research (WRAIR) Human Subject Protection Board (HSPB) (KEMRI/SERU/CCR/0053/3385: WRAIR#1743A).

### GC isolation and species verification

Samples were taken only after the informed consent and assent process was completed and the study subject was enrolled into the parent STI surveillance study. Depending on the sex of the subjects, urethral swabs for males or of cervical swabs for females were collected. Microscopy examination of gram stained swabs followed by laboratory culture of swab material to identify gram negative diplococci was used to screen specimens for further culture, isolation and identification of *Neisseria gonorrhoeae*. Plates containing selective GC medium were inoculated with genital swabs and transported in a CO_2_ enriched environment to the Centre for Microbiology Research laboratory (CMR), located at the KEMRI headquarter for isolation, identification and confirmation of *N. gonorrhoeae* before antimicrobial susceptibility testing (AST) was undertaken. Once the inoculated transport medium reached the CMR laboratory, it was incubated at 37 °C in an incubator enriched with 5% CO_2_ for 48 h and examined daily for any growth.

To obtain pure isolates for Antibiotic Susceptibility Testing (AST), gram negative, catalase and oxidase positive *Neisseria* colonies from the transport medium were grown in a newly prepared non selective GC medium base supplemented with IsoVitalex™, BD. Antimicrobial susceptibility testing was done on the pure isolates following confirmation using API NH (Biomerieux). Minimal inhibitory concentrations (MICs) to selected antibiotics were determined using the E-test strip (A B, Biodisk, Sweden) method. Pure isolates were stocked on Trypticase soy broth with 20% glycerol and stored at − 80 °C. For genomic characterization of multi-drug resistant GC isolates, the frozen isolates were thawed, inoculated on Modified Thayer-Martin (GC agar, BD containing vancomycin, nystatin, colistin and trimethoprim lactate) and incubated at 37 °C in 5% CO_2_ for 18 to 24 h. Plates with typical GC growth were further subjected to oxidase and catalase biochemical tests.

### DNA extraction and quantitation

Genomic DNA from confirmed GC positive cultures was extracted using QIAamp DNA Mini Kit (Qiagen, Hilden, Germany) according to the manufacturer’s instructions. The quality and quantity of genomic DNA were determined by Qubit® dsDNA HS Assay using Qubit 3.0 fluorometer, (Thermo Fisher Scientific Inc. Wilmington, Delaware USA) according to the manufacturer’s instructions. Quantified DNA was stored in a − 20 °C freezer prior to sequencing.

### Whole-genome sequencing and assembly

#### Library preparation and sequencing

Libraries were prepared from 5 μg of genomic DNA of each sample. The DNA was processed by Illumina Nextera XT sample preparation kit (Illumina Inc. San Diego, CA, USA) and uniquely indexed using Illumina Nextera XT Index Kit (Illumina Inc. San Diego, CA, USA). The libraries were purified and normalized using Agencourt AMPure XP beads (Beckman Coulter, Beverly, Massachusetts). All recovered elutes were pooled in equal volumes. DNA concentration of the pool was determined using Qubit ds DNA HS Assay (Thermo Fisher Scientific Inc. Wilmington, Delaware USA). Paired end reads were generated on Illumina MiSeq platform (Illumina, San Diego, CA, USA) using a paired-end 2 × 250 bp protocol.

### De novo assembly

Sequence raw reads were assembled through the assembly pipeline (version1.2) available from the Center for Genomic Epidemiology (CGE) (https://cge.cbs.dtu.dk/services) which is based on the Velvet algorithms for de novo assembly of short reads [32]. Assembled contigs were aligned against FA1090 (GenBank Accession number AE0049969) *N. gonorrhoeae* reference genome using Mauve [33]. Sequence reads for this study are deposited on National Centre for Biotechnology Information (NCBI) SRA Accession SRP154258.

### Genome-wide single nucleotide polymorphism phylogeny

To determine the genetic diversity between the study isolates, additional 38 draft genome sequences of *N. gonorrhoeae* strains obtained from varied geographical regions were downloaded from NCBI (https://www.ncbi.nlm.nih.gov/) and included in the analysis. Single Nucleotide Polymorphisms (SNPs) were identified using the CSI phylogeny pipeline available on Centre for Genome Epidemiology (CGE) (http://www.genomicepidemiology.org) [34]. Raw sequence reads and the additional draft genome sequences were mapped to *Neisseria gonorrhoeae* NCCP11945 (GenBank accession number CP001050) reference genome, using BWA version v.0.7.17 [35]. SNPs from each genome were called using SAMTools v.1.7 [36], and concatenated into a single fasta alignment. The concatenated sequences were used for constructing a maximum likelihood tree using RaxML with generalized time reversible (GTR) model [37]. The tree was visualized by using FigTree version 1.4.3 [38]

### Identification of antimicrobial resistance determinants

Annotation of the whole genome to determine fluoroquinolone resistance was performed with Rapid Annotation using Subsystem Technology (RAST) (http://rast.nmpdr.org) in Pathosystems Resource Integration Center version 3.5.7 (PATRIC) (https://www.patricbrc.org) [39] and different antibiotic determinants were saved into separate feature groups. Reference GyrA (GenBank Accession number U08817) and ParC (GenBank Accession number U08907) were downloaded from the NCBI website https://www.ncbi.nlm.nih.gov. Mutations in the *gyrA*, *parC*, *porB* and *mtrR* genes and proteins were determined using Bioedit sequence alignment editor version 7.0.5 [40].

### Statistical analysis

Wilcoxon Mann-Whitney statistical test was conducted in GraphPad Prism V7.0.4 (GraphPad Software) [41]. Statistical comparisons were two tailed and were performed with the significance level set at *P* < 0.05.

## Abbreviations

AST: Antibiotic Susceptibility Testing
CGE: Center for Genomic Epidemiology
CMR: Centre for Microbiology Research
EUCAST: European Committee on Antimicrobial Susceptibility Testing
GC: *Neisseria gonorrhoeae*
KEMRI: Kenya Medical Research Institute
MIC: Minimum Inhibitory Concentration
MSM: Men who have Sex with Men
QRDR: Quinolone Resistant Determining Region
SRA: Sequence Read Archive
STI: Sexually Transmitted Infection
